# Re-evaluating a historic cohort of sinonasal and skull base mucoepidermoid carcinoma: an institutional experience

**DOI:** 10.1186/s13000-024-01466-5

**Published:** 2024-03-01

**Authors:** Chunyan Hu, Lan Lin, Ming Ye, Yifeng Liu, Qiang Huang, Cuncun Yuan, Ji Sun, Hui Sun

**Affiliations:** 1 Department of Pathology, Eye & ENT Hospital, Fudan University, 2600 Jiangyue Road, Shanghai, 201112 China; 2 Department of Pathology, Fudan University Shanghai Cancer Center, Shanghai, 200032 China; 3 Department of Otorhinolaryngology, Eye & ENT Hospital, Fudan University, Shanghai, 200031 China; 4 Department of Radiotherapy, Fudan University Shanghai Cancer Center, 270 Dongan Road, Shanghai, 200032 China

**Keywords:** Sinonasal tract and skull base, Mucoepidermoid carcinoma, *MAML2*, Hyalinizing clear cell carcinoma, *EWSR1::ATF1*, *DEK::AFF*

## Abstract

**Aims:**

Primary mucoepidermoid carcinomas (MECs) of the sinonasal tract and nasopharynx are rare entities that represent a diagnostic challenge, especially in biopsy samples. Herein, we present a case series of MECs of the sinonasal and skull base and its mimics to evaluate the clinicopathological and molecular characteristics in order to avoid misdiagnosis.

**Methods:**

We reviewed the pathology records of patients diagnosed from 2014 to 2022. Thirty MECs were consecutively diagnosed during that period.

**Results:**

Based on morphological and fluorescence in situ hybridization (FISH) analyses, 30 tumors originally diagnosed as MECs were separated into *MAML2* fusion-positive (7 cases) and *MAML2* fusion-negative groups (23 cases), in which 14 tumors were positive for the *EWSR1::ATF1* fusion; these tumors were reclassified to have hyalinizing clear cell carcinoma (HCCC). The remaining nine *MAML2* FISH negative cases were reconfirmed as squamous cell carcinoma (SCC, 3 cases) which showed keratinization and high Ki-67 expression; *DEK::AFF2* carcinomas (2 cases), in which *DEK* gene rearrangement was detected by FISH; and MECs as previously described (4 cases) with typical morphological features. Including 7 *MAML2* rearrangements tumors, 11 MEC cases had a male-to-female ratio of 4.5:1, and 6 tumors arose from the nasopharyngeal region, while 5 tumors arose from the sinonasal region. The prognosis of this series of salivary gland-type MECs was favorable.

**Conclusions:**

Our study confirmed that HCCC runs the risk of being misdiagnosed as MEC in the sinonasal tract and nasopharynx, particularly with biopsy specimens. Careful histological evaluation with supporting molecular testing can facilitate pathological diagnoses.

## Introduction

Mucoepidermoid carcinoma (MEC) is the most common salivary gland malignancy, especially in the major salivary glands. Salivary gland-type tumors of MECs arising from the sinonasal tract and nasopharynx were first described in the literature by at least the 1970s [1]. In earlier studies, MEC in this anatomical region was seldom observed by general surgical pathologists, accounting for < 0.1% of primary malignant neoplasms and resulting in potential diagnostic dilemmas [2]. Clinically, the usual symptoms are related to the location of the primary tumor and are nonspecific, including nasal bleeding and obstruction, headache, facial numbness, tinnitus or hearing loss, deafness and diplopia [3]. The etiopathogenesis, treatment and prognosis of salivary gland-type malignant tumors are still uncertain. Morphologically, analogous to their salivary gland counterparts, MECs are characterized by a mixture of cell types including mucinous cells, intermediate cells and epidermoid cells, and are presumed to originate predominantly from the mucosal mucoserous glands. Studies from the salivary gland literature have shown that up to 75% to 80% of MECs harbor gene fusions involving *MAML2*, and the diagnostic utility of this translocation status has been recognized, especially when assessing small biopsies and tumors with overlapping histopathology [4]. With increasing evidence, *MAML2* translocation has been traditionally associated with low- and intermediate-grade tumors.

MECs sometimes share common morphological features with hyalinizing clear cell carcinoma (HCCC), because both tumors can contain mucin and clear cells. HCCC is characterized by small monomorphic cells with pale eosinophilic cytoplasm that are similar to intermediate MEC cells. Therefore, the differential diagnosis between HCCC and MEC can sometimes be very difficult [5]. Since endoscopic surgery often serves as the primary treatment for sinonasal tract and nasopharynx tumors, and since all specimens for pathological examination are fragmented, obtaining the correct diagnosis of MEC seems particularly challenging. Fortunately, more than 80% of HCCC tumors harbor this unique *EWSR1::ATF1* fusion and *EWSR1* rearrangement, which distinguishes HCCC from other salivary gland tumors [6–8]. Primary sinonasal tract or nasopharynx MECs are uncommon tumors that are frequently misdiagnosed, resulting in inappropriate clinical management. Due to its rarity, studies analyzing sinonasal and skull base MEC are mostly limited to case reports and small case series in the English literature. Specifically, there is no large comprehensive evaluation of primary sinonasal or skull base MECs with respect to their molecular genetics and differential diagnosis.

In this study, we revaluated the histopathological features of MEC patients with an original diagnosis of MEC and the morphological mimics of these tumors in the sinonasal tract and skull base. Furthermore, we investigated the differential diagnosis and molecular characteristics of salivary gland-type tumors in this uncommon location.

## Materials and methods

### Patients and samples

In our cohort, we obtained formalin-fixed paraffin-embedded (FFPE) material from patients with an initial diagnosis of MEC primarily arising from the sinonasal and nasopharynx regions (30 cases) who underwent surgical resection at the Department of Otolaryngology of the Affiliated Eye Ear Nose and Throat (EENT) Hospital, Fudan University from 2014 to 2022. FFPE tissue blocks were obtained from the archives. Two pathologists reviewed the specimens and designated representative blocks identified from hematoxylin/eosin (HE) stained sections. Written informed consent was obtained from all participants in the study. Ethical approval was granted by the Institutional Review Committee of the Eye and ENT Hospital of Fudan University.

### Immunohistochemistry

IHC staining was performed using a BenchMark Autostainer (Ventana Medical Systems, Tucson, USA) according to the manufacturer’s protocol. The retrieved FFPE blocks were cut into 3 μm-thick sections. IHC was performed for most primary antibodies, including P63 (4A4, 1:100 dilution; Gene Tech, Shanghai), P40 (GR006; Gene Tech), CK7 (OV-TL12130; Maixing, Shanghai), CK5/6 (MX040 Maixing, Shanghai), and P16 (GM501; Gene tech), and proliferation index (Ki-67, Clone GM001; Gene Tech, Shanghai, China). Appropriate positive and negative controls were run with each batch. Briefly, the sections were deparaffinized and then pretreated with Cell Conditioner 1 at 95 °C for 76 min and a peroxidase inhibitor for 4 min. Staining was performed according to the manufacturers’ recommendations for each antibody. Secondary antibodies were added to all slides with HRP multimer and incubated for 8 min. The extent of IHC expression was quantified in quartiles as previously reported [9]: 0, negative; 1 + , 1–25% positive cells; 2 + , 26–50% positive cells; 3 + , 51–75% positive cells; and 4 + ,76–100% positive cells. The percentage of p16 stained tumor cells was evaluated, and at least 70% of the tumor cells were nuclear and cytoplasmic expression as considered positivity according to the expert consensus opinion of the College of American Pathologists (CAP) [10]. For Ki-67, the absolute percentage of positive tumor cells was recorded. Periodic acid-Schiff (PAS) and Alcian blue staining were performed according to standard protocols.

### Fluorescence in situ hybridization

Fluorescence in situ hybridization (FISH) was performed on FFPE sections using a commercially available *MAML2* dual color break apart probe (Zytovision), Vysis *EWSR1* Dual Color Break Apart FISH Probe (Abbott Molecular Inc.) and *DEK* Dual Color Break Apart FISH Probe (Abbott Molecular Inc., Des Plaines IL, USA) to assess *MAML2, EWSR1* and *DEK* gene translocation, respectively, for clinical use. For each slide, 50 randomly selected nonoverlapping tumor cell nuclei were examined by a pathologist. Separate green and orange signals in the nucleus were considered rearrangements and were considered positive if > 20% of the tumor cell nuclei showed break-apart signals. In addition, the *EWSR1::ATF1* Fusion-Translocation Probe Kit (Vysis LSI *EWSR1* Dual-Color Dual-Fusion Translocation; Abbott Molecular) was used according to the manufacturer’s instructions. The fusion translocation was indicated by one separate red signal, one separate green signal, and two yellow fusion signals. FISH for gene rearrangement was considered positive if > 20% of the tumor cell nuclei showed fusion signals.

### In situ hybridization (ISH)

EBV-encoded small RNA was analyzed through in situ hybridization of *EBER* genes 1 and 2 with an inform *EBER* probe (Ventana Medical Systems, Tucson, USA) according to the manufacturer’s protocol. The intended target is the early RNA transcripts of EBV accumulate in the nucleus of EBV-infected cells, as evaluated by a blue reaction that is localized to EBV-infected nuclei. Positive hybridization was defined as punctuate or diffuse signals in the nucleus of the tumor cells. An appropriate positive control was used in all cases.

### Human papillomavirus (HPV) detection and genotyping

As previously described [9], HPV was detected using polymerase chain reaction (PCR) to amplify the L1 gene in conjunction with reverse dot blot (RDB) analysis to identify the HPV subtypes for clinical use. This kit offers a simple testing strategy involving a membrane chip that can detect infections from multiple HPV subtypes, including 18 high-risk types (HPV16, 18, 31, 33, 35, 39, 45, 51, 52, 53, 56, 58, 59, 66, 68, 73, 82 and 83) and 5 low-risk types (HPV 6, 11, 42, 43 and 44). β-Globin was used as an internal positive control.

## Results

### Clinical features

These cases were originally diagnosed with MEC and were tested for *MAML2* by FISH, and these cases were separated into *MAML2* fusion–positive (7 cases) and *MAML2* fusion-negative groups (23 cases) groups. *MAML2* fusion–negative patients were selected for the detection of *EWSR1* rearrangement and *EWSR1::ATF1* fusion by FISH. Fourteen HCCC cases were initially misdiagnosed with MEC via biopsy. Additionally, *DEK* break-apart FISH showed positive signals in two cases. Four tumors originally diagnosed as MEC were *MAML2* gene rearrangement negative and were classified as one high-grade tumor and three low- or intermedia-grade tumors according to their typical morphological features. The other three *MAML2* fusion–negative tumors were reclassified as SCC, in which one patient had a history of sinonasal SCC after carefully checking the patient's medical records. All three of these cases exhibited keratinization with high Ki-67 expression. The clinical features of the 11 reidentified MEC cases are summarized in Table 1. There were 9 males and 2 females, and the median age was 53 years (range: 31–82 years). Most tumors were unilateral (n = 10) and involved the nasal cavity alone (n = 1), or the nasopharynx alone (n = 2), the other eight patients showed the combination of involvement of the nasal cavity, nasopharynx, paranasal sinuses, orbit and skull base. Only one patient showed involvement of the nasal cavity, hard palate, infratemporal fossa, and ethmoid sinus. Clinically, the most common symptoms were associated with a nonspecific nasal obstruction (n = 7) and epistaxis (n = 6). Endoscopic transnasal resection was performed for 9 patients, and two patients underwent initial excision followed by wide excision and maxillectomy surgery. Four patients received postoperative radiotherapy after surgery (200 cGy each) and chemotherapy (cisplatin). Follow-up data were available for 11 patients ranging from 24 to 144 months, with an average of 59 months (median 48 months); 5 patients were alive and well at their last follow-up, six patients developed recurrence with an average follow-up of 73 months (range 37–144 months), and case 9 had multiple lymph node metastases.

**Table 1:** Clinical features of Sinonasal tract and Skull base mucoepidermoid carcinoma (MEC)

Case no	Age	Sex	Main symptoms	Involved sites	TNM	Treatment	Follow-up	Outcome
1	54	M	Epistaxis, headache	Right sphenoid sinus, nasopharynx, slope	T3N0M0	Endoscopic surgery	68 months	Alive with disease
2	31	M	Epistaxis	Right nasopharynx	T1N0M0	Endoscopic surgery	43 months	Alive without disease
3	65	M	Nasal dorsum swelling, lacrimal discharge, nasal obstruction	Right nasal cavity, maxillary and ethmoid sinus, orbit, facial	T4aN0M0	Endoscopic surgery + RT,CT	67 months	Alive without disease
4	53	M	Nasal obstruction, epistaxis	Right sinus, nasopharynx, orbit, skull base	T3N0M0	Endoscopic surgery + RT,CT	96 months	Alive with disease
5	31	F	Tinnitus	Left nasopharynx, sphenoid sinus	T3N0M0	Endoscopic surgery	42 months	Alive with disease
6	65	F	Left tinnitus	Double nasal cavity, hard palate, infratemporal fossa, ethmoid sinus	T3N0M0	Maxillectomy surgery	144 months	Alive with disease
7	53	M	Left nasal obstruction, epistaxis	Left nasal cavity, nasopharynx top	T1N0M0	Extended resection	24 months	Alive without disease
8	70	M	Left nasal obstruction, epistaxis	Left nasal cavity, maxillary sinus, ethmoid sinus, infratemporal fossa	T4aN0M0	Endoscopic surgery + RT	48 months	Alive without disease
9	50	M	Right nasal obstruction, hyposmia	Nasopharynx, Sphenoid sinus, ethmoid sinus	T3N3M0	Endoscopic surgery + lymph node dissection	51 months	Alive with disease
10	52	M	Nasal obstruction, epistaxis	Nasopharynx	T1N0M0	Endoscopic surgery + RT	30 months	Alive without disease
11	82	M	Nasal obstruction	Right nasal cavity	T2N0M0	Endoscopic surgery	37 months	Alive with disease

*CT* Chemotherapy, *RT* Radiation therapy;

A subgroup of HCCC patients and the clinical features are summarized in Table 2. Most of these tumors arose from the nasopharynx, and the patients had a male-to-female ratio of 1:1. The clinical symptoms were nonspecific, and included nasal obstruction and epistaxis. Endoscopic transnasal resection was performed in 12 patients, and one patient underwent received maxillectomy surgery. Moreover, four patients received postoperative chemoradiotherapy, and one patient received radiation therapy immediately after diagnosis. Two of the 14 patients died of the disease with follow-up ranging from 48 to 102 months and one patient was lost to follow-up.

**Table 2:** Clinical features of Sinonasal tract and Skull base hyalinizing clear cell carcinoma (HCCC)

Case no	Age	Sex	Main symptoms	Involved sites	TNM	Treatment	Follow-up	Outcome
1	31	F	Nasal obstruction	Nasopharynx	T1N0M0	Endoscopic surgery	37 months	Alive without disease
2	39	F	Nasal obstruction	Nasopharynx	T1N0M0	Endoscopic surgery	55 months	Alive without disease
3	35	M	Right nasal obstruction	Right nasopharynx, intracranial	T4bN0M0	Endoscopic surgery + RT,CT	102 months	Dead with disease
4	53	M	Left ear tightness	left nasopharynx	T2N0M0	Endoscopic surgery + RT,CT	74 months	Alive with disease
5	32	M	Left facial swelling	Right maxillary sinus	T3N0M0	Maxillectomy surgery	48 months	Alive without disease
6	64	M	Nasal epistaxis	Roof of nasopharynx	T1N0M0	Endoscopic surgery	37 months	Alive without disease
7	32	F	Right nasal epistaxis	Right nasopharynx	T4N0M0	Endoscopic surgery	37 months	Alive without disease
8	73	F	Nasal bleeding	Roof, lateral and posterior wall of nasopharynx	T2N0M0	Endoscopic surgery + RT,CT	190 months	Alive with disease
9	33	F	Nasal obstruction, runny nose	Nasopharynx	T2N0M0	Endoscopic surgery	65 months	Alive with disease
10	50	F	Sore throat	Left nasopharynx, intracranial	T4N0M0	Endoscopic surgery	/	/
11	67	M	Nasal bleeding	Left nasopharynx, nasal cavity and cervical lymph node	T2N0M0	Endoscopic surgery + RT,CT	60 months	Dead with disease
12	43	M	Left nasal obstruction	Nasopharynx	T1N0M0	Endoscopic surgery	65 months	Alive without disease
13	65	M	Nasal bleeding	Nasopharynx	T1N0M0	Endoscopic surgery	84 months	Alive without disease
14	70	F	Nasal bleeding	Nasopharynx	T1N0M0	Biopsy + RT	68 months	Alive without disease

*CT* Chemotherapy, *RT* Radiation therapy, / Lost to follow-up

### Morphological features

**Fig. 1:**
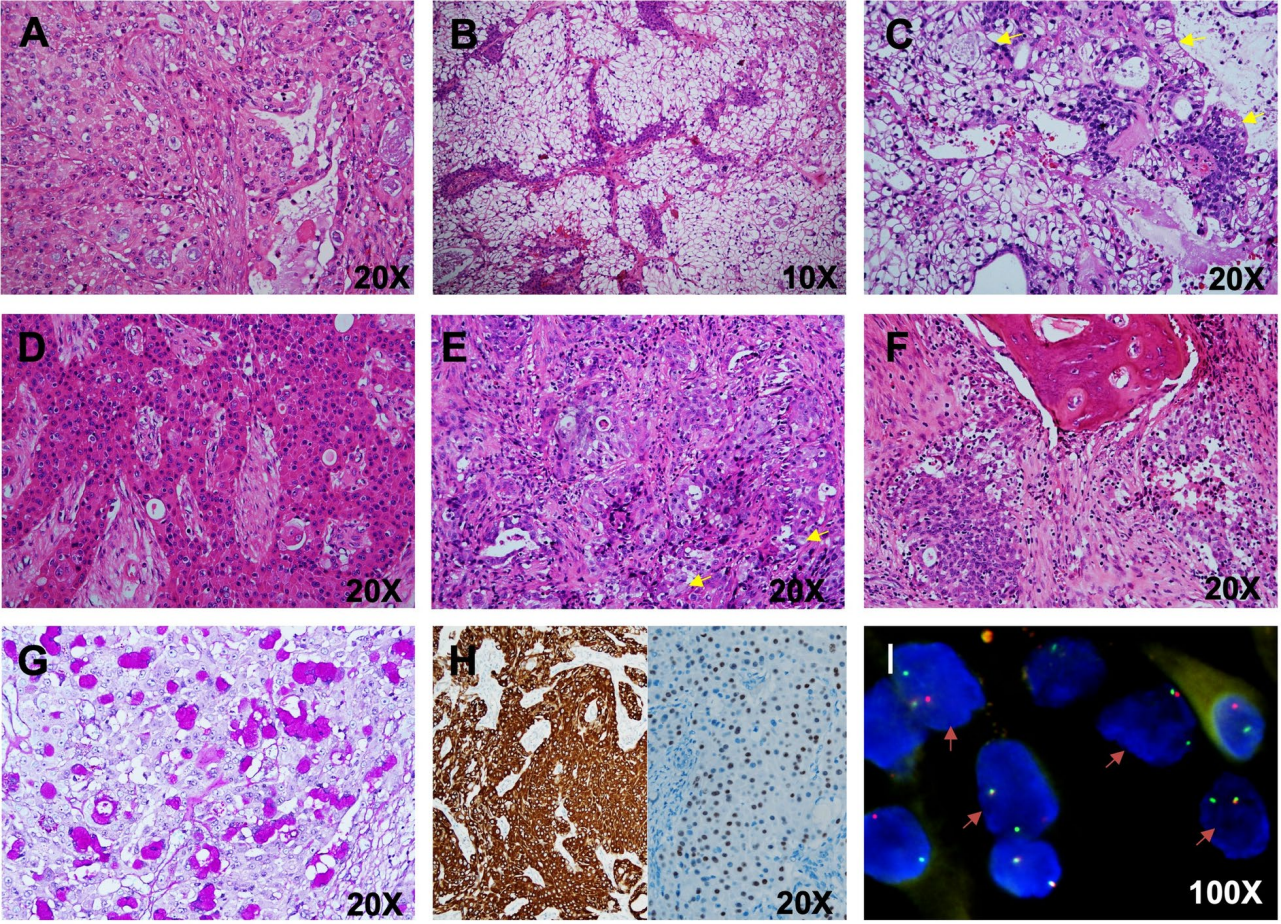
Histopathologic, immunohistochemical and molecular pathology findings of sinonasal tract and skull base mucoepidermoid carcinoma (MEC). Low-grade MEC consisting of lobules and cysts of epidermoid, mucin, and intermediate cells (**A**), clear cell variant of MEC with solid lobules of clear cell forming fibrovascular stroma (**B**), small hyperchromatic nuclei, occasional mucus-producing cells (arrows) (**C**), neoplastic oncocytic cells showing abundant, finely granular cytoplasm, and a moderate degree of nuclear atypia (**D**), high-grade MEC showing squamous epithelioid with large nuclei, a few clear mucinous cells (arrows) and intermediate cells (**E**), bone invasion (**F**), periodic acid–schiff staining demonstrated the mucus-producing cells (**G**), diffuse expression of CK7 and intense nuclear staining for p63 in tumor cells (**H**), FISH revealed *MAML2* rearrangement in 7 cases (**I**)

**Fig. 2:**
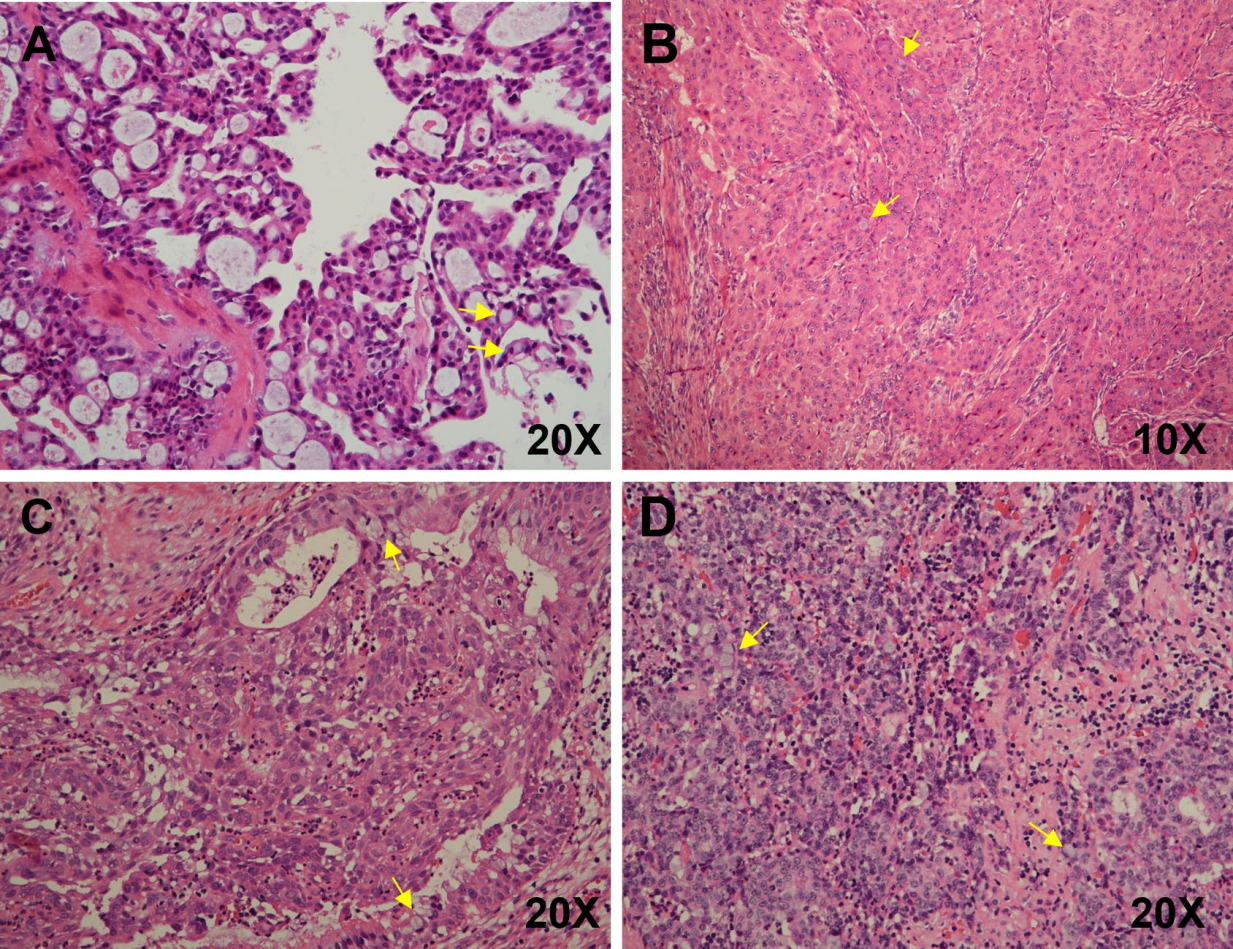
Histopathologic findings of *MAML2* fusion-negative mucoepidermoid carcinoma (MEC). Histopathology showing the features of low-grade MEC, demonstrating cystic spaces lined by epidermoid cells, intermediate cells and mucus-producing cells (arrows) (**A**), Intermedia-grade MEC with oncocytic, occasional mucus-producing cells (arrows) (**B**), high-grade MEC showing squamous epithelioid with large nuclei, occasional gland formation or mucin production (arrows) (**C**), intermedia-grade MEC with a moderate degree of nuclear atypia epidermoid cells, mucous cells (arrows) and inflammatory cells infiltration (**D**)

Due to the anatomic site of involvement, specific macroscopic tumor features were not well-described. The tumors were pale, yellow to reddish tan, and showed multiple fragments in most cases. Microscopically, all MECs were usually composed of epidermoid cells, mucocytes and intermediate cells in varying proportions, forming cystic structures filled with mucin (Fig. 1A). One tumor was composed predominantly of clear cells (90%) and appeared to be a clear cell variant of MEC (CCMEC). The tumor cells were arranged in nests and islands, which were surrounded by fibrovascular stroma and presented with papillary structures. Very small amounts of intermediate (less than 10%) and mucous cells (less than 1%) were recognized (Fig. 1B-C). Two tumors (18.2%) predominantly had nests of oncocytic cells and were classified as oncocytic variants of MEC (OMEC). The percentage of oncocytic cells ranged from 75% to greater than 90% (Fig. 1D). The typical oncocytes showed centrally placed nuclei with granular eosinophilic cytoplasm and a low nuclear-to- cytoplasmic ratio. Using the Armed Forces Institute of Pathology (AFIP) criteria [11], three tumors were classified as high-grade MECs, and bone invasion was documented in one high-grade MEC (Fig. 1E-F). The oncocytic variants were categorized as 1 low grade and 1 intermediate grade, and the clear cells were categorized as intermediate grade according to the criteria. Necrotic areas were observed in 4 of the 11 cases (36.4%). Four cases (36.4%) with *MAML2* fusion–negative MECs were included in this study, and these MECs, comprising one low-grade MEC, two intermediate-grade MECs and one high-grade MEC, had some typical features of MECs. All 4 tumors had numerous mucus-secreting, epidermoid and intermediate cells forming variably sized cystic structures. The epidermoid component of the lesions showed a spectrum that ranged from low grade (Fig. 2A) to intermediate grade to poorly differentiated high grade (Fig. 2B-D).

**Fig. 3:**
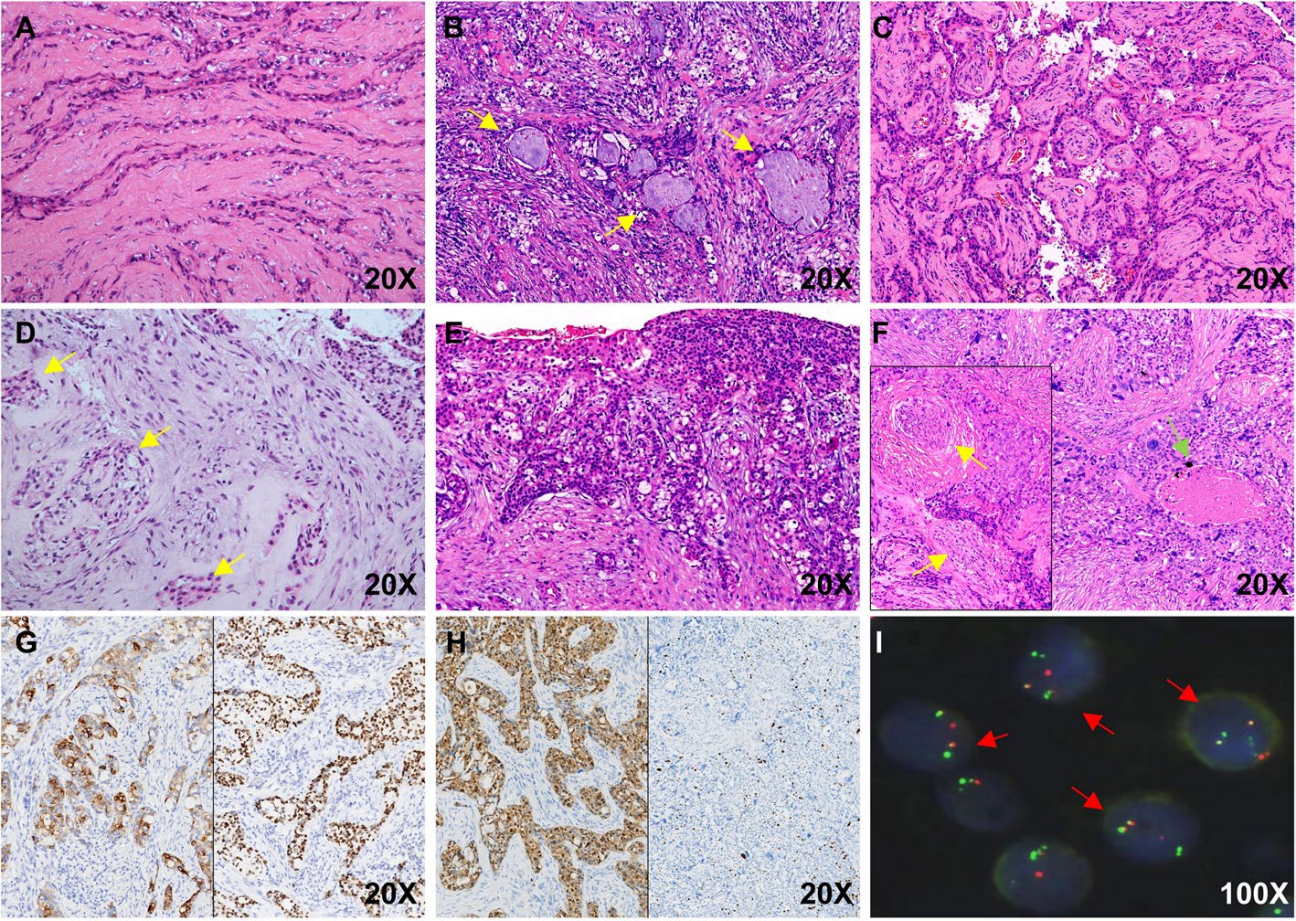
Histopathologic, immunohistochemical and molecular pathology findings of sinonasal tract and skull base hyalinizing clear cell carcinoma (HCCC). Note the cords and trabeculae growth patterns with prominent hyalinized collagen (**A**), proportions nests and cords of bland cells with clear cytoplasm with occasional gland-like formation or mucin production (arrows) (**B**), papillary structure of the fibrovascular axis (**C**), predominant clear cells, eosinophilic cells and their mixture in various proportions (arrows) (**D**), HCCC exhibited surface epithelial involvement (**E**), varying degrees of necrosis (green arrows) and nerve invasion (yellow arrows) (**F**), neoplastic cells strongly and diffusely expressed CK7 (left) and p63 (right) (**G**), p16 positive (left) and two cases showed relatively high Ki-67 (right) (**H**), FISH revealed *EWSR1-ATF1* fusion in all HCCC cases (**I**). (× 1000)

The subgroup of 14 patients was reclassified as sinonasal and skull base HCCC which are characterised by features similar to those of their salivary counterparts. Most tumors were unencapsulated and infiltrative with solid sheets, nests, cords, and trabeculae growth patterns. One of 14 HCCC cases (7.1%) exhibited surface epithelial involvement. Characteristic hyalinized acellular collagen bundles were easily recognized, and the tumor cells exhibited clear eosinophilic components with occasional gland-like formation or mucin production (Fig. 3A-D). Varying degrees of necrosis were found in five cases, and two tumors had high expression levels of Ki-67 (10–20%), one of which involved nerve invasion (Fig. 3E). In case 11, the patient underwent endoscopic surgery and chemoradiation therapy and was followed-up for 48 months with left cervical lymph node metastasis. There was no obvious clear cytoplasm or gland formation, but there was a papillary structure, which was also observed in the other two cases (Fig. 3F).

**Fig. 4:**
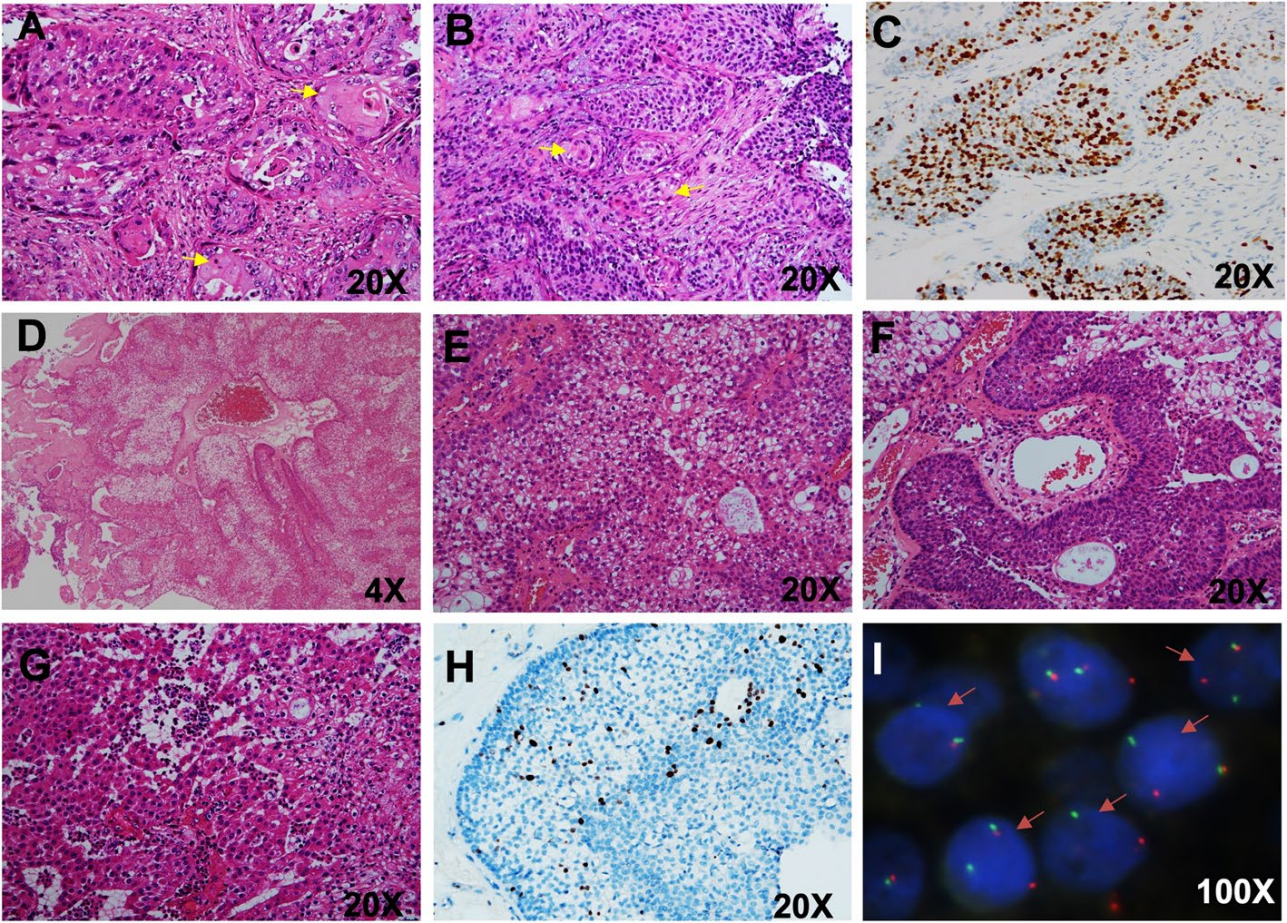
Histopathologic, immunohistochemical and molecular pathology findings of sinonasal tract and skull base SCC and *DEK::AFF2* carcinomas. Keratinizing SCCs with intercellular bridges and obvious keratin pearl formation (**A-B**), and high expression levels of Ki-67 (**C**); *DEK::AFF2* carcinomas demonstrated a mix of exophytic and endophytic growth, with broad papillary fronds (**D**), immature transitional-type epithelial cells monotonous and round to oval with delicate chromatin and prominent nucleoli (**E**), a blunted papillae and pushing pattern of invasion into stroma as interconnecting ribbons (**F**); a dense population of tumor-infiltrating neutrophils (**G**); Ki-67 index was approximately 5% (**H**), The *DEK* gene rearrangement was confirmed by FISH (**I**)

The above three SCC tumors showed more or less keratinization, intercellular bridges and high Ki-67 expression (Fig. 4A-C). The other two cases were changed to *DEK::AFF2* carcinomas, which share similar histopathological features with papilloma-like growth. Specifically, one tumor generally displayed peculiar acantholytic changes, which manifested as a loss of cellular cohesion. The other tumor had more than 50% distinct clear cells. Other histological features included immature transitional-type epithelial cells, absence of overt keratinization and prominent tumor-infiltrating neutrophils or stromal lymphocytes (Fig. 4D-G).

### Immunohistochemical fingdings

Table 3 and Table 4 summarize the immunohistochemical and molecular findings, respectively, for the 11 MEC and 14 HCCC patients. For the 11 patients with MECs, special staining of extracellular mucin was highlighted with Alcian blue and PAS (Fig. 1G). Most of the MECs and HCCC cases expressed CK7, CK5/6, P63 (Fig. 1H) and P40 (Fig. 2G). Of the investigated HCCC cases, two (14.3%) had relatively high Ki-67 levels (10–20%), and two were p16 positive according to the expert consensus opinion of the CAP [10] (Fig. 2H). The new entity of *DEK::AFF2* carcinomas was diffusely positive for CK5/6, p40, and p63, and negative for CK7. The Ki-67 index was very low (< 5%) (Fig. 3H).

**Table 3:** Immunohistochemical findings, fluorescence in situ hybridization (FISH) and In situ hybridization (ISH) tests of 11 patients with mucoepidermoid carcinoma (MEC)

Case no	Grade (AFIP)	Grade (Brandwein)	Necrosis	Histological features	P63 (%)	CK7 (%)	CK5/6 (%)	Ki-67 (%)	P16 (%)	HPV (ISH)	MAML2 (FISH)	EBER (ISH)
1	Intermedia-grade	High-grade	+	conventional MEC	4 +	3 +	4 +	3%	-(40%)	-	+	-
2	Low-grade	Intermedia-grade	-	conventional MEC	1 +	4 +	4 +	3%	-(50%)	-	+	-
3	High-grade	High-grade	-	conventional MEC	4 +	1 +	4 +	70%	-(0)	-	+	-
4	High-grade	High-grade	+	conventional MEC	2 +	2 +	3 +	focal 50%	-(0)	-	+	-
5	Low-grade	Low-grade	-	conventional MEC	4 +	3 +	4 +	2%	-(10%)	-	+	-
6	Intermedia-grade	Intermedia-grade	-	**clear cell MEC**	1 +	4 +	2 +	5%	-(10%)	-	+	-
7	Low-grade	Intermedia-grade	-	**eosinophilic MEC**	2 +	4 +	4 +	4%	-(30%)	+	+	-
8	High-grade	High-grade	+	conventional MEC	2 +	1 +	4 +	focal 20%	-(20%)	-	-	-
9	Intermedia-grade	Intermedia-grade	-	conventional MEC	4 +	1 +	4 +	8%	-(60%)	-	-	-
10	Intermedia-grade	Intermedia-grade	-	**eosinophilic MEC**	2 +	4 +	4 +	6%	-(10%)	-	-	-
11	Low-grade	Intermedia-grade	+	conventional MEC	2 +	3 +	3 +	15%	+ (80%)	-	-	-

**Table 4:** Immunohistochemical findings, fluorescence in situ hybridization (FISH) and in situ hybridization (ISH) tests of 14 patients with hyalinizing clear cell carcinoma (HCCC)

Case no	Clear cell (%)	Nerve invasion	Necrosis	P40	P63	CK7	CK5/6	Ki-67 (%)	P16 (%)	EWSR1 (FISH)	EWSR1::ATF1 (FISH)	EBER (ISH)	HPV (ISH)
1	50%	-	-	4 +	4 +	2 +	4 +	3%	-(40%)	+	+	-	-
2	10%	-	-	4 +	4 +	1 +	4 +	2%	-(30%)	+	+	-	-
3	5%	+	+	2 +	3 +	4 +	4 +	focal10%	+ (90%)	+	+	-	-
4	10%	-	+	1 +	1 +	4 +	2 +	2%	-(30%)	+	+	-	-
5	90%	-	-	1 +	1 +	4 +	4 +	1%	-(0)	+	+	-	-
6	5%	-	-	3 +	2 +	4 +	4 +	2%	-(10%)	+	+	-	-
7	5%	-	-	4 +	4 +	1 +	4 +	3%	-(20%)	+	+	-	-
8	10%	-	+	4 +	4 +	4 +	4 +	4%	-(30%)	+	+	-	-
9	40%	-	+	4 +	4 +	4 +	4 +	4%	+ (80%)	+	+	-	-
10	10%	-	-	2 +	3 +	0	3 +	1%	-(20%)	+	+	-	-
11	10%	-	+	2 +	1 +	1 +	1 +	10–20%	-(30%)	+	+	-	-
12	20%	-	-	3 +	4 +	4 +	4 +	3%	-(10%)	+	+	-	-
13	20%	-	-	4 +	4 +	4 +	4 +	4%	-(10%)	+	+	-	-
14	50%	-	-	2 +	4 +	3 +	4 +	3%	-(20%)	+	+	-	-

### HPV, EBV infection and molecular findings

All MECs and HCCCs were tested for HPV, and one MEC patient was identified as high risk for HPV16 positivity and showed p16 negativity and *MAML2* FISH rearrangement. Among the 14 HCCC and 3 SCC patients investigated, none were positive for HPV infection (all types combined) or negative for EBV infection according to ISH. Seven of the 11 MEC cases (63.6%; 3 low-grade, 2 intermediate-grade and 2 high-grade) had FISH rearrangement, including one case of CCMEC and one case of OMEC (Table 3 and Fig. 1I). In addition, the *EWSR1::ATF1* fusion was identified for both the *EWSR1* split and fusion FISH probes (Fig. 2I). Importantly, *DEK* break-apart FISH and histological features reconfirmed the diagnosis of *DEK-AFF2* carcinoma (Fig. 3I).

## Discussion

MEC in the sinonasal and skull base is a very rare entity (< 0.1%) at our hospital. In accordance with the findings of a previous report, MECs in these uncommon locations share similar histopathological features with salivary MECs, which are characterized by varying amounts of epidermoid cells, intermediate cells, and mucinous cells arranged in cystic and solid growth patterns in variable proportions. Clinically, MEC of the salivary gland usually presents in children and young adults, with a peak incidence occurring from the 5th to 6th decades had a female predilection [12, 13]. Wolfish et al. reported that sinonasal tract MEC usually presents in patients aged 15–75 years (mean: 52.7 years), and that there does not seem to be a sex difference [2]. In our study, we found that males seemed to be more frequently affected (male/female ratio of 4.5:1), with a median age of 53 years (range: 31–82 years), which may be attributed to the special anatomical location of the disease. The symptoms were nonspecific and related to the site of involvement. During the past decade, *MAML2* fusion has been a frequent chromosomal rearrangement observed in salivary gland MECs [14].Several studies have shown that up to approximately 80% of salivary MECs are fusion-positive, which preferentially occurs in patients with low-grade tumors with an excellent prognosis [15]. To our knowledge, the frequency of *MAML2* rearrangement has not been previously reported in sinonasal or nasopharyngeal MECs. Recent studies have shown that tumors with *MAML2* rearrangements are more likely to be classified as low grade [16]. In our study, seven of the cases in our series (63.6%; 3 low-grade, 2 intermediate-grade and 2 high-grade) harbored *MAML2* gene rearrangements, providing further evidence of the decreased rate of *MAML2* translocations in sinonasal and nasopharynx MEC. Furthermore, four of 11 MEC cases (36.4%) were *MAML2* fusion-negative, comprising one low-grade, two intermediate-grade and one high-grade tumor. Our results indicated that there was only one low-grade MEC case showing *MAML2* fusion-negative while *CRTC1/3-MAML2* fusion positivity seemed to be more common in low-grade MECs, which may be the main difference between those four fusion-negative cases and the seven fusion-positive cases. Although the lack of *MAML2* rearrangement raises the question of the nature of these MEC tumors, all four cases presented more or less typical morphological features of MEC and had an immunohistochemical profile similar to that of *MAML2* fusion cases.

Most of the *MAML2* fusion-negative MEC in the sinonasal tract and nasopharyngeal location were HCCCs (n = 14), which partially shares histologic features with MECs, including clear cells, eosinophilic cells, cystic components and mucin-rich cells. Furthermore, the presence of clear cells and oncocytic cells in MECs further complicates diagnosis, especially when these cell types predominate. Not surprisingly, HCCC runs the risk of being misdiagnosed as MEC when the tumor classification is based on morphological features alone, especially when assessing biopsy specimen. Typically, characteristic hyalinized acellular collagen bundles were easily recognized within HCCCs, while glandular formation was slightly more frequent in MECs. We also found that 3 of 14 (21.4%) HCCCs presented with papillary structures, and one of 14 (7.1%) HCCC cases exhibited surface epithelial involvement. This finding is in accordance with Bishop et al. [17]. Although perineural and bone invasion was more frequent in HCCC, we found that only one case presented nerve invasion. This may be due to our limited case series in this uncommon location. HCCC is a low-grade carcinoma that is readily cured by surgical excision, but histological scoring revealed two of the HCCC cases to be intermediate-grade tumors according to Taka et al*.*’s scoring principle, and these two patients died of the disease during follow-up [18]. Our study further showed that salivary gland-type HCCCs arising from the sinonasal tract and nasopharynx were not significantly related to sex and were less likely to develop recurrence.

Considering the studies showing that high-risk HPV may play an etiologic role in MEC of the minor salivary gland [19], we suspected that HPV could be a potential.

pathogenic factor for sinonasal and nasopharynx MEC and found that one case in our series contained HPV DNA. It is indicated that p16 negative certainly not transcriptionally active high-risk HPV. Additionally, even though all of the HCCCs demonstrated some degree of p16 staining, none were found to harbor high-risk HPV DNA. This finding is in line with previous research [17]. The other morphologic mimics of MEC, including SCC, and *DEK::AFF2* fusion-associated papillary SCC, were renamed “*DEK::AFF2* carcinomas” [20]. SCC of the sinonasal tract and nasopharynx is an important differential diagnosis, particularly in cases with prominent clear cell features. However, SCC typically lacks three cell types and sometimes show keratinization with high Ki-67 index. A novel *DEK-AFF2* fusion was recently reported in nonkeratinizing SCC of the sinonasal region and skull base, characterizing histologic features including immature transitional-type epithelial cells growing as papillary structures and broad ribbons deep into the stroma with a striking infiltrate of inflammatory cells [21]. Two *DEK::AFF2* carcinomas in our cases share similar histopathological features with previous reports, which showed peculiar acantholytic change by loss of cellular cohesion and distinct clear cells that resemble clear cell variant of MEC extremely. Overall, the distinctive molecular and histologic features of *DEK::AFF2* carcinomas suggest that they represent a unique entity in the sinonasal region. *DEK* is an oncogene that plays a key role in hetero- chromatin regulation and is consistently upregulated in various cancer types including both HPV-positive and HPV-negative head and neck SCC [22]. *AFF2* is a transcriptional regulator that is best known in the germline setting as a cause of intellectual disability, yet *AFF2* itself has never previously been implicated in cancer [23]. With the recognition of a larger cohort of skull base and sinonasal carcinomas that harbor *DEK::AFF2* fusions and confirmation that a subset lacks other known oncogenic mutations, our study solidifies the role of this unique genetic event as the key driver of these tumors.

In our series, six of 11 MEC patients experienced recurrence and none of the patients died of the disease, whereas 3 of 14 HCCC patients experienced recurrence, and two died of the disease. One of the three SCC patients died of the disease. These results emphasize the importance of applying strict criteria to diagnose MEC because of the different prognostic implications. In a recent study on head and neck MEC, 46 cases were originally identified as MEC while 24 of these cases were reclassified into a more aggressive tumor type [24]. MEC of the sinonasal tract has a low risk for metastases and tends to be locally aggressive [25]. The separation of differential diagnoses and variant forms of MEC is critical. In recent years, surgical resection has been the mainstay treatment for sinonasal tract MEC and the most significant predictor of survival, with a dramatic difference in 5-year survival from 72.9% for surgically treated patients to 23.5% for patients who did not receive surgery [25]. Additionally, adjuvant radiation and negative margins were associated with improved survival in patients. Among the 3 high-grade cases in our series, adjuvant radiotherapy-chemotherapy was administered and 2 were alive without disease at the last follow-up, indicating that adjuvant therapy may provide benefits for local disease control. The role of targeted therapies for locally aggressive MECs remains to be explored.

## Conclusion

In summary, we presented originally diagnosis of MEC from 2014 to 2022 in our institution which was reclassified by morphology and molecule. The two most common pathologic entities in the non-MEC groups were HCCC and SCC, focusing on histologic, immunohistochemical, and FISH findings. Our study highlighted the importance of combining histologic evaluation and molecular testing in the identification of salivary gland-type tumors arising in the sinonasal or nasopharynx and its imitations.

## Data Availability

No datasets were generated or analysed during the current study.
